# Analysis of SOX2-Regulated Transcriptome in Glioma Stem Cells

**DOI:** 10.1371/journal.pone.0163155

**Published:** 2016-09-26

**Authors:** Arlet M. Acanda de la Rocha, Hernando López-Bertoni, Elizabeth Guruceaga, Marisol González-Huarriz, Naiara Martínez-Vélez, Enric Xipell, Juan Fueyo, Candelaria Gomez-Manzano, Marta M. Alonso

**Affiliations:** 1 The Health Research Institute of Navarra (IDISNA), Pamplona, Spain; 2 Program in Solid Tumors and Biomarkers, Foundation for the Applied Medical Research, Pamplona, Spain; 3 Department of Pediatrics, University Hospital of Navarra, Pamplona, Spain; 4 Hugo W Moser Research Institute at Kennedy Krieger, Baltimore, Maryland, United States of America; 5 Department of Neurology, The Johns Hopkins School of Medicine, Baltimore, Maryland, United States of America; 6 Bioinformatics Unit, Center for Applied Medical Research, Pamplona, Spain; 7 Brain Tumor Center, University of Texas MD Anderson Cancer Center, Houston, Texas, United States of America; University of Alabama at Birmingham, UNITED STATES

## Abstract

**Introduction:**

Glioblastoma is the most malignant brain tumor in adults and is associated with poor survival despite multimodal treatments. Glioma stem-like cells (GSCs) are cells functionally defined by their self-renewal potential and the ability to reconstitute the original tumor upon orthotopic implantation. They have been postulated to be the culprit of glioma chemo- and radio-resistance ultimately leading to relapse. Understanding the molecular circuits governing the GSC compartment is essential. SOX2, a critical transcription regulator of embryonic and neural stem cell function, is deregulated in GSCs however; the precise molecular pathways regulated by this gene in GSCs remain poorly understood.

**Results:**

We performed a genome-wide analysis of SOX2-regulated transcripts in GSCs, using a microarray. We identified a total of 2048 differentially expressed coding transcripts and 261 non-coding transcripts. Cell adhesion and cell-cell signaling are among the most enriched terms using Gene Ontology (GO) classification. The pathways altered after SOX2 down-modulation includes multiple cellular processes such as amino-acid metabolism and intercellular signaling cascades. We also defined and classified the set of non-coding transcripts differentially expressed regulated by SOX2 in GSCs, and validated two of them.

**Conclusions:**

We present a comprehensive analysis of the transcriptome controlled by SOX2 in GSCs, gaining insights in the understanding of the potential roles of SOX2 in glioblastoma.

## Introduction

Glioblastoma is the most common and deadly primary brain tumors in adults and despite multiple treatments, the survival of glioma patients remains poor, with a median time between 12–15 months [1–3]

Glioblastoma contains a subpopulation of tumor propagating stem-like cells, known as glioma stem-like cells (GSCs) [4], which display the ability to self-renew, to differentiate into distinct lineages and to efficiently initiate and propagate tumors in xenografts models that recapitulate the phenotypic characteristics of the initial tumor from which they were derived [5,6]. Moreover, GSCs have been shown to increase resistance to radio-and chemotherapy [7,8], explaining in part the poor overall survival despite multiple treatments.

SOX2, a member of the SRY gene family, is a key transcription factor in the regulation of stemness properties, and it is essential in early embryonic development [9]. SOX2 has been reported to be deregulated in several human cancers [10–12] including glioblastoma where is over expressed due to several mechanisms such as amplification and promoter hypomethylation [13]. SOX2 is enriched in human-derived GSCs where it sustains stemness, migration, invasion and maintenance of tumorigenicity [13,14]. Although SOX2 response program in a glioblastoma cell line has been analyzed [15], to the best of our knowledge, an exhaustive analysis of SOX2-regulated molecular circuitries in GSCs has not been performed. Deciphering the molecular circuitries controlled by SOX2 in GSCs will provide insights about glioma development, biology and possible novel molecular therapies.

Besides coding genes, long non-coding RNAs (lncRNAs) are an emerging class of RNAs with no functional protein-coding ability that consists of more than 200 nucleotides [16]. Recent discoveries have proven that they play important roles regulating gene expression and function. These non-coding RNAs actively participate in many pathological processes in human malignancies [17–19] including cancer where a number of lncRNAs have been shown to act as oncogenes or tumor suppressors [20]. Recently, different groups published a signature of lncRNAs with aberrant expression in glioblastoma [21] and a set of prognostic lncRNAs that could have clinical implications in the sub-classification of this disease [22]; though the functional effect of lncRNAs in glioblastoma is not well understood.

Given that SOX2 is predominantly expressed in the GSCs compartment, which plays prominent roles in driving the growth, treatment resistance and recurrence of glioblastoma, the elucidation of the transcriptome and the molecular pathways involved in the generation and maintenance of this recalcitrant population is critical to understand the molecular underpinnings of glioblastoma malignancy. The aim of this work was to characterize the transcriptome regulated by SOX2 in GSCs. We set out to describe not only the coding genes but also the lncRNAs, which have been shown to play predominant roles in cancer. In this study we present a comprehensive analysis of the transcriptome controlled by SOX2 in GSCs, gaining insights in the understanding of the potential roles of SOX2 in glioblastoma.

## Materials and Methods

### Cell Lines, culture and transfection

Neurosphere cultures (GSC11 and GSC23), a kind gift of Dr. Lang at UT MD Anderson Cancer Center, were established from acute cell dissociation of human glioblastoma surgical specimens and maintained in Dulbecco's modified Eagle's medium/nutrient mixture F12 supplemented with B27 (Invitrogen, Carlsbad, CA), epidermal growth factor, and basic fibroblast growth factor (20 ng/mL each; Sigma-Aldrich, St Louis, MO).

To inhibit SOX2 expression, transient transfection assays were performed using two commercially available, specific siRNA against human SOX2 (si-SOX2, s13295 and s13294, Ambion) and a non-targeting control siRNA (si-scramble) (Ambion) in four independent experiments. The siRNA transfections were performed according to the manufacturer's instructions using Lipofectamine 2000 (Invitrogen). The cells were then cultured for 72 h after transfections and subjected to different analysis.

### RNA extraction and Real Time PCR analysis

Total cellular RNA was isolated from the cultured cells using a Trizol reagent (Ambion) according to the manufacturers' protocols. For lncRNAs analysis, total RNA was subjected to DNase I treatment to digest the DNA. RNA quantity and quality were measured by NanoDrop ND-1000. The RNA samples were then reversely transcribed into cDNA using the Taqman miRNA Reverse Transcription Kit (Applied Biosystems) according to the manufacturer's instructions. Real Time PCR (RT-PCR) was performed using the Sybr Green Fast Master Mix (Applied Biosystems) in the ABI 7700 sequence detection system (Applied Biosystems, Foster City, CA). The quality of the products was controlled by the melting curve. Transcript levels were normalized against human GAPDH. Transcripts expression levels relative to GAPDH were calculated using the ddCt method. Primers for lncRNA detection and quantification were designed at Universal ProbeLibrary Assay Design Center (http://www.roche-applied-science.com/). All primer sequences are listed below (Tables 1 and 2):

**Table 1:** Primers of lncRNAs for qRT-PCR.

lncRNA position	TCONs	Forward Primer	Reverse Primer
chr19:28281401–28284848	TCONS_00027256	GCCCAAAGTTTGATTTCTCG	CGAGGTCTAACCCAGGTGTG
chr11:121899032–121899389	TCONS_00020142	GCTGAGCCTTCCATGAAAAT	GTGCAAATCACTCCAGTCACA

**Table 2:** Primers of genes for qRT-PCR.

Gene	Forward Primer	Reverse Primer
GAPDH	AGCCACATCGCTCAGACAC	GCCCAATACGACCAAATCC
SOX2	AGCTCGCAGACCTACATGAA	CCGGGGAGATACATGCTGAT
PLP1	ACCTATGCCCTGACCGTTG	TGCTGGGGAAGGCAATAGACT
COL2A1	TGGACGCCATGAAGGTTTTCT	TGGGAGCCAGATTGTCATCTC
ATP8B1	ACGACATTTGACGAGGATTCTC	GGTTTTGTTCTGGTTCAACAGC
PPP1R1B	CAAGTCGAAGAGACCCAACCC	GCCTGGTTCTCATTCAAATTGCT
CMTM5	GGAGGACCACATCCGCTAGAT	CCAGGGAGTGGAAGCAGAT
GALNT14	CACTGCTGGTGTATTGCACG	CGGATCAGATGCGTAGGGG
F11R	GTGCCTACTCGGGCTTTTCTT	GTCACCCGGTCCTCATAGGAA
SYT4	ATGGGATACCCTACACCCAAAT	TCCCGAGAGAGGAATTAGAACTT
SLC18A1	GTGGTGGTATTCGTCGCTTTG	CCGAGGTGCAGAGAAGAGT
ITLN2	GCAGGGCAACAAAGCAGACTA	CAGGGCGCTGTTTCTCCAA

### Immunoblotting Assay

For the western blot assay, cells were lysed in RIPA buffer (Triton and PBS) for 30 min on ice. Samples containing identical amounts of protein (30 μg) were resolved in a 12% polyacrylamide gel, transferred to polyvinylidene membranes, and blocked in 5% nonfat milk in phosphate-buffered saline/Tween-20. Membranes were incubated with the following antibodies: SOX2 (Cell Signaling, Danvers, MA) and α-Tubulin (Sigma-Aldrich) using 1:1000 dilution. The membranes were developed according to the protocol for enhanced chemiluminiscence from Perkin Elmer.

### Microarray expression analysis

Total RNA was isolated from scrambled and SOX2-siRNA GSC11 cells using Trizol extraction. RNA was purified by the QIAGEN RNAeasy mini kit (QIAGEN) according to the manufacturer´s protocol. One-color Cy3 RNA labeling, array hybridization to Agilent SurePrint G3 8 × 60 K Human Gene Expression Arrays (Agilent Technologies), data collection, and analysis were performed at the Bioinformatics Unit (Fundación para la Investigación Médica Aplicada, CIMA, Pamplona, Spain). Normalization of microarray data was performed using quantile algorithm. After quality assessment a filtering process was carried out to eliminate low expression probe sets. Applying the criterion of an expression value greater than 64 in 2 samples of at least one of the experimental conditions, 40986 probe sets were selected for statistical analysis. LIMMA (Linear Models for Microarray Data) [23] was used to find out the probe sets that showed significant differential expression between experimental conditions. Genes were selected as significant using a B statistic cut off B>0. Data processing and statistical analyses were performed with R and Bioconductor [24]. The microarray data from this study are publicly available at Gene Expression Omnibus (http://www.ncbi.nlm.nih.gov/geo) under accession number GSE79302.

### Functional group analysis

In this study we applied Gene Ontology (GO) analysis to find the primary function of the differential expression of mRNAs regulated by SOX2, using online software DAVID (Database for Annotation, Visualization and Integrated Discovery, http://david.abcc.ncifcrf.gov/). GO analysis can organized genes into hierarchical categories (Gene Ontology Consortium). To find out the significant pathway of the differential genes participating we performed gene regulatory network analysis using Ingenuity Pathway Analysis (IPA) software (http://www.ingenuity.com), which can integrate gene-expression data with other molecular databases to facilitate the development of new and more complete pathway maps. Fisher's exact test was used to select the significant GO categories. The threshold of significance was defined by P value with a cut-off set in 0.05.

### Statistical analysis

Experimental data are represented as the mean ± SD of three biologic replicates and were compared using Student's t-test. Significant P-values are indicated with asterisks as follows: *P < 0.05, **P < 0.01.

## Results

### Transcripts regulated by SOX2

**Fig 1:**
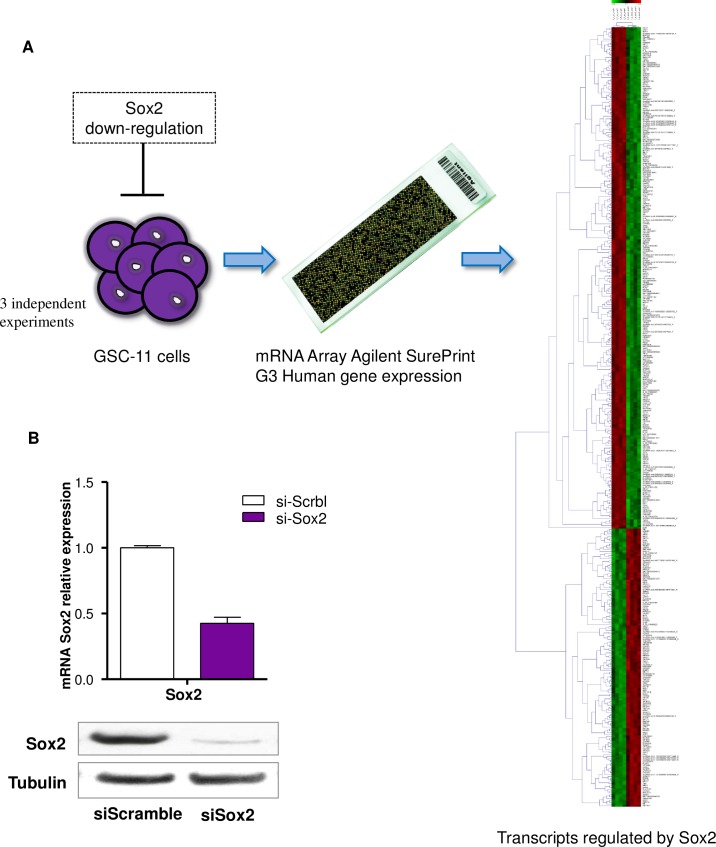
Transcripts regulated by SOX2. (A) Schematic representation of the research design employed to uncover the SOX2 transcriptome in GSC11 cells. (B) qRT-PCR and western blot confirmation of SOX2 inhibition in GSC11 cells after 72h of si-SOX2 or si-Scramble (si-Scrbl) transfection. SOX2 relative mRNA levels are presented as 2^-ΔΔ^Ct standardized with their constitutive gene GAPDH. Each bar represents the mean ± SD. For western blot tubulin was used as housekeeping control and shown as a representative blot of four independent experiments.

Since SOX2 is a key driver in the maintenance of the GSCs phenotype and therefore in the perpetuation of this devastating tumor we down-regulated the expression of this gene in the GSC11 cell line in four independent experiments, using a SOX2 specific siRNA (Fig 1A). The efficiency of SOX2 knockdown was assessed by real-time PCR and western blot (Fig 1B) and was also confirmed in our array results. Microarray data identified a total of 2048 differentially expressed coding transcripts and 261 non-coding transcripts (B value >0) (Fig 1A and S1 Table).

### SOX2 controls a wide spectrum of protein-coding genes and pathways in GSCs

**Fig 2:**
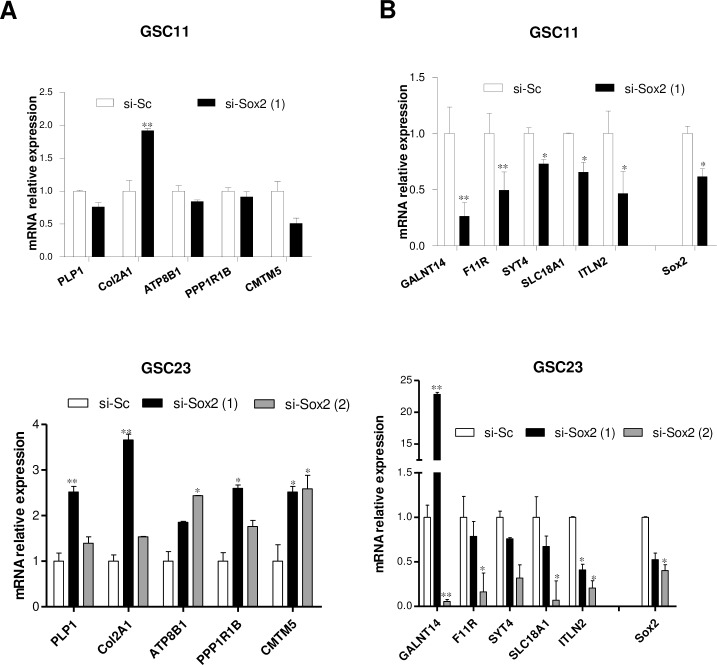
**Analysis by qRT-PCR of the top 5 (A) up- and (B) down-regulated coding transcripts in SOX2 downmodulated GSC11 and GSC23 cells.** Total RNA was extracted after 72h of si-Sc or si-SOX2 transfection in GSC11 and GSC23 cells. In GSC23 cells we used two different siRNAs against human SOX2, si-SOX2 (1) is referred to s13295 and si-SOX2 (2) is referred to s13294 from Ambion. Values are normalized to GAPDH and each bar represents the mean ± SD.

To further narrow the coding transcripts data a cut-off 1 logarithmic fold difference between SOX2 knockdown and scrambled GSC11 cells was set, identifying 35 up-regulated and 100 down-regulated genes, which suggest that SOX2 act primarily as a transcriptional activator. In Table 3 we showed the top-10 up or down-regulated protein coding-genes, and select the top 5 candidates of each group for further validation by qRT-PCR using the GSC11 and GSC23 cell lines. We confirmed the observed microarray expression changes in 5 out of 5 down-regulated coding-genes (Fig 2B) and in one out of 5 up-regulated coding genes in GSC11 cells (Fig 2A). Regarding GSC-23 cells, we down-modulate SOX2 expression using two different siRNAs against human SOX2, and we validate the expression of 5 out of 5 up-regulated coding-genes (Fig 2A) and of 4 out of 5 down-regulated coding genes (Fig 2B), partially validating the microarray results.

**Table 3:** The top 10 up- and down-regulated genes in Sox2-downmodulated GSC-11 cells, organized by logFC.

GeneName	logFC	P.Value	B
PLP1	2,443	4,73E-05	2,488
COL2A1	2,122	2,18E-05	3,281
ATP8B1	1,954	1,50E-06	5,943
PPP1R1B	1,925	7,27E-06	4,393
CMTM5	1,702	1,12E-04	1,586
ELMO1	1,66	5,97E-05	2,246
ITIH5L	1,555	6,67E-05	2,133
IGFBP5	1,522	1,82E-06	5,759
SCARNA9	1,498	7,10E-05	2,067
SCARNA17	1,435	4,46E-06	4,879
GALNT14	-3,006	0,000299	0,569
F11R	-2,824	1,13E-07	8,308
SYT4	-2,664	2,61E-06	5,405
SLC18A1	-2,335	4,01E-06	4,984
ITLN2	-2,329	3,57E-07	7,288
RASEF	-2,294	9,38E-07	6,39
GADD45G	-2,231	3,55E-08	9,277
CYP26A1	-2,056	7,66E-10	11,938
KRTAP21-1	-2,028	0,00021	0,94
PNLIPRP2	-1,992	0,000495	0,042

To understand the significance of differential gene expression, bioinformatics analysis related to Gene Ontology (GO) Classification and pathway analysis were performed. GO classifications using the DAVID web tool and pathway analysis using Ingenuity Pathway Analysis (IPA) was performed. For these analysis, gene lists were classified based upon decreased (logFC < -1) or increased (logFC > 1) expression relative to control and analyzed altogether as a single list.

**Fig 3:**
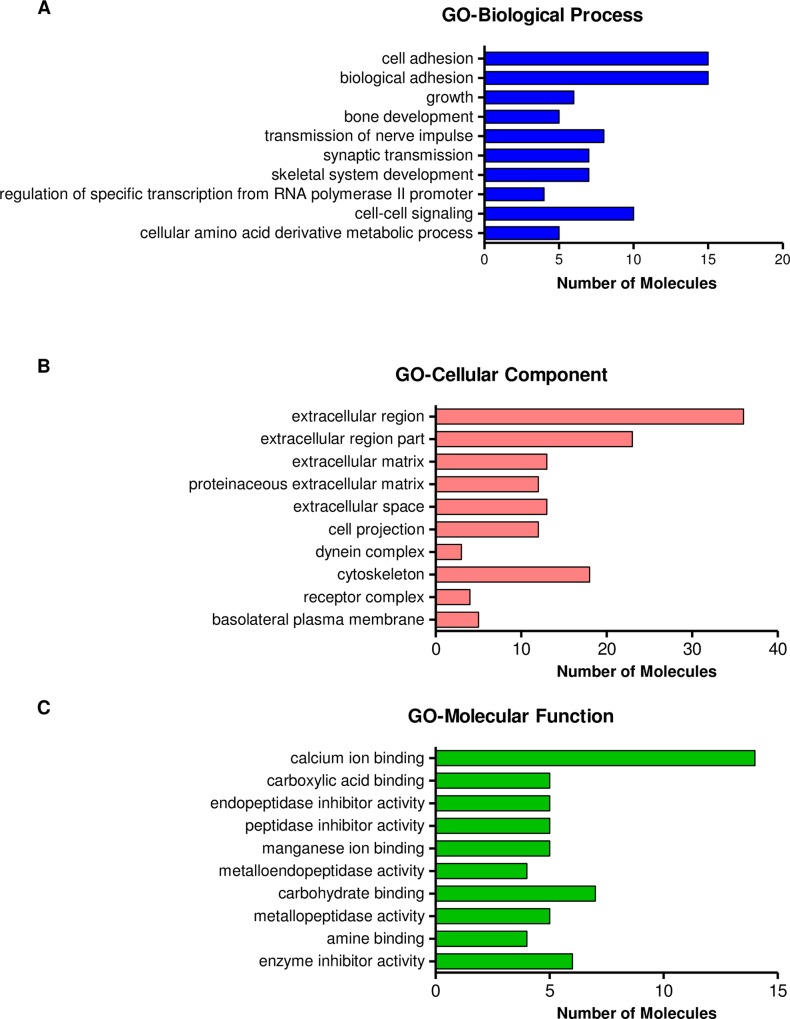
Top-10 GO Biological Processes analysis of protein-coding genes regulated by SOX2 in GSC11 cells. Bar chart represents classification of GO Biological Processes, Cellular Component or Molecular Function as determined by DAVID web tool. Bars represent the number of genes in the specified category, organized by p-value.

Enrichment analysis of GO categories including biological process (BP), molecular function (MF), and cellular component (CC) were obtained using DAVID web tool (Fig 3). We observed the highest enrichment in the categories related to “cell adhesion”, “biological adhesion”, “cell-cell signaling”, “extracellular region” and “calcium ion binding”.

We used IPA analysis to undercover the canonical pathways regulated by SOX2 in GSCs. Our results showed 13 pathways significantly altered (Table 4). Most of them related with amino-acid metabolism pathways, such as “histamine biosynthesis”, where histamine is an important regulator of numerous physiological processes including neurotransmission in the central nervous system (CNS) [25]; “L-cysteine degradation process” where cystathionine γ-lyase (CTH) activity has been related with glioblastoma treatment [26] and “serotonin receptor signaling pathway” being serotonin an important neurotransmitter in the CNS during neuronal development [27]. Other enriched pathways were “hematopoiesis from multipotent stem cells”, where KITLG has been reported to regulate neoplastic processes such as growth and invasion [28]; apoptosis [29] and cell adhesion [30]. Role of JAK2 in Hormone-like Cytokine Signaling stood out because GHR (growth hormone receptor) and IRS1 (insulin receptor substrate 1) has been linked with glioma progression [31,32]. A well-characterized pathway frequently altered in tumors is the NOTCH signaling cascade, which was also enriched in our analysis. The NOTCH pathway is a conserved intercellular signaling route that has been implicated in different developmental processes. Interestingly, NOTCH pathway is deregulated in human glioblastoma and plays a key role in maintaining the growth, the undifferentiated state of glioma cells and tumorigenesis [33–35]. The integrated analysis of SOX2 enriched canonical pathways revealed the link between this transcription factor and multiple cellular processes such as amino-acid metabolism and intercellular signaling cascades, like NOTCH pathway.

**Table 4:** List of top-13 canonical pathways identified by IPA software.

Pathway	-log(p-value)	Ratio	Molecules
Glycine Betaine Degradation	2,63E+00	2,50E-01	DMGDH,PIPOX
Hepatic Stellate Cell Activation	2,38E+00	4,42E-02	LY96,COL2A1,COL22A1,IGFBP5,COL28A1
Histamine Biosynthesis	2,03E+00	1,00E+00	HDC
L-cysteine Degradation II	2,03E+00	1,00E+00	CTH
Triacylglycerol Degradation	2,02E+00	1,25E-01	PNLIPRP2,CES1
Retinol Biosynthesis	2,02E+00	1,25E-01	PNLIPRP2,CES1
Hematopoiesis from Multipotent Stem Cells	1,73E+00	5,00E-01	KITLG
Cysteine Biosynthesis/Homocysteine Degradation	1,73E+00	5,00E-01	CTH
Role of JAK2 in Hormone-like Cytokine Signaling	1,61E+00	7,69E-02	GHR,IRS1
Serotonin Receptor Signaling	1,58E+00	7,41E-02	SLC18A1,HTR1D
Phenylethylamine Degradation I	1,56E+00	3,33E-01	AOC3
Notch Signaling	1,35E+00	5,56E-02	HES5,HEY1
Lysine Degradation V	1,34E+00	2,00E-01	PIPOX

This selection is organized by the negative logarithm of p-values (Fisher Test), calculated by IPA ([-Log (0.05) = 1.3]).

**Fig 4:**
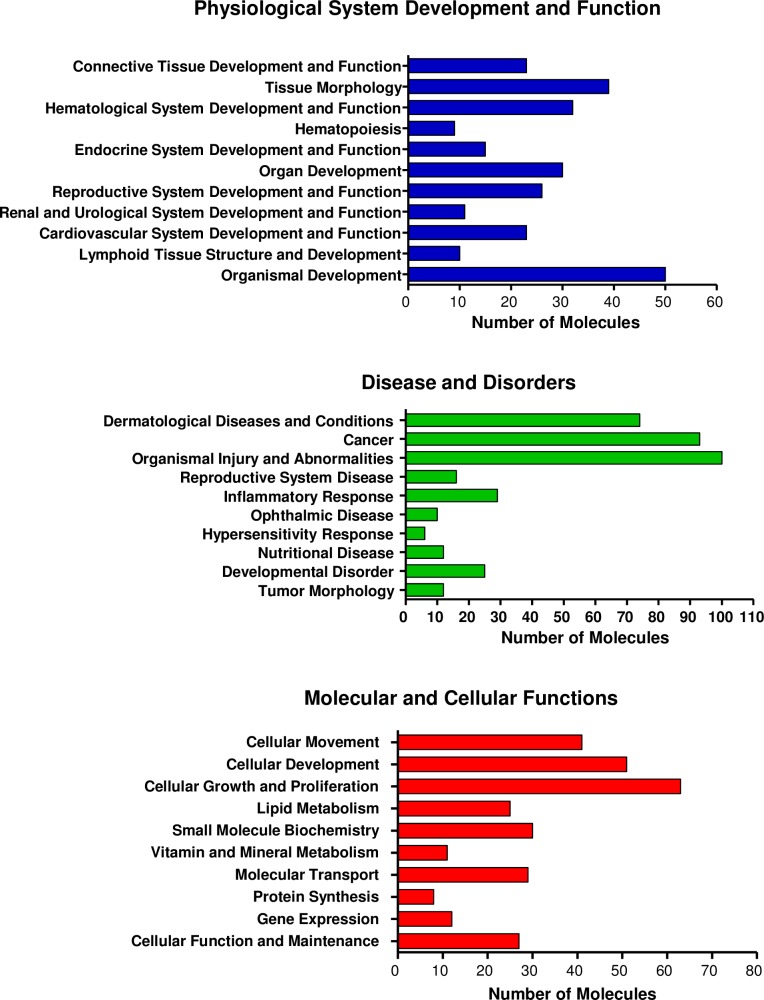
10-Top Bio Functions categories altered following SOX2 inhibition. The categories listed are Physiological System Development and Function, Molecular and cellular Functions and Disease and Disorders, identified using IPA software. Bars represent the number of genes in the specified category, organized by p-value.

The IPA analysis also showed the most relevant biological functions and diseases in our data set. The most significant bio-functions altered following SOX2 down-modulation are showed in Table 5. The set of SOX2-associated genes were assigned mainly to the following networks: “cancer”, “organismal injury and abnormalities”, “cellular movement”, “tissue morphology”, “cellular development” and “hematopoiesis”. Interestingly, most of these networks involved very well-known functions of SOX2 such as morphology determination [36], development [37] and cellular proliferation and migration in glioma [13,13]. Fig 4 shows the most relevant selection of bio-function categories: disease and disorders, molecular and cellular functions and physiological system development and function, obtained by using IPA software organized by p-value.

**Table 5:** The top ten significant Bio-Functions altered following Sox2 down-modulation in the GSC11 cell line.

Category	p-value	Number of Targets
Dermatological Diseases and Conditions	1,63 x 10^−08^–9,35 x 10^−03^	74
Cancer	2,93 x 10^−08^–9,35 x 10^−03^	93
Organismal Injury and Abnormalities	2,93 x 10^−08^–9,35 x 10^−03^	100
Cellular Movement	3,98 x 10^−06^–9,35 x 10^−03^	41
Connective Tissue Development and Function	1,71 x 10^−05^–9,35 x 10^−03^	23
Tissue Morphology	1,71 x 10^−05^–9,35 x 10^−03^	39
Reproductive System Disease	2,52 x 10^−05^–9,35 x 10^−03^	16
Cellular Development	2,72 x 10^−05^–9,35 x 10^−03^	51
Hematological System Development and Function	2,72 x 10^−05^–9,35 x 10^−03^	31
Hematopoiesis	2,72 x 10^−05^–9,35 x 10^−03^	9

The p-value range indicates the p-values of the various pathways and processes belonging to that category. The number of targets indicates the total number of genes associated with the functional category.

These results established a signature of protein coding-genes regulated by SOX2 in GSCs with biological functions relevant to glioblastoma growth and maintenance of its malignant phenotype. The tight overlap between the existing literature and our enrichment analysis highlights the robustness of our results and predicts that this approach will be an excellent discovery platform to identify novel SOX2 targets.

### SOX2–regulated non-coding RNAs in GSC

**Fig 5:**
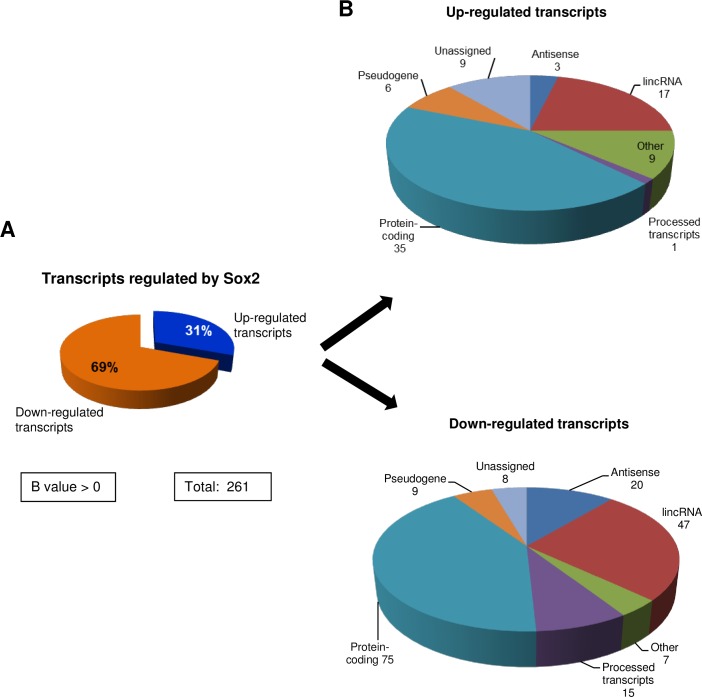
SOX2 regulated non-coding transcripts. (A) A total of 261 transcripts were found differentially expressed (B > 0), which were distributed in 80 upregulated and 181 downregulated transcripts. **(B)** Biotype distribution of the differentially expressed transcripts following SOX2 down-modulation in GSC11 cells.

Reprogramming transcription factors, including SOX2, have been shown to regulate both coding and non-coding RNAs [38]. LncRNAs are emerging as key regulators of biological processes and disease [39] therefore, seems reasonable to hypothesize that SOX2 will regulate this class of genes as well. The strength of our data-sets allowed us to identify potential non-coding transcripts differentially expressed (B value > 0) regulated by SOX2 in GSCs. After biotype distribution analysis we identify protein coding RNAs (44% for up-regulated and 41% for down-regulated), while the rest were classified as different types of non-coding transcripts. Out of the total number of transcripts differentially expressed we identify 80 upregulated and 181 down-regulated and we classify them as intergenic RNAs, antisense, processed transcripts, transcripts derived from pseudogenes and unassigned transcripts (Fig 5). The transcripts classified as “others” correspond to transcripts derived from miRNAs, rRNAs, sense-overlaping and sense intronic transcripts. The lncRNA annotation was performed with the Bioconductor package ChIPpeakAnno [40] and using Gencode v19 as reference [41]. The gene type corresponding to the gene that overlaps with the lincRNA locus was assigned to each lincRNA. Table 6 shows the top 25 non-coding transcripts regulated by SOX2 in GSCs.

**Table 6:** List of the top 25 non-coding transcripts regulated by Sox2, organized by B value.

Probe	GeneName	Classification	logFC	B
A_19_P00320471	lincRNA:chr9:2535671–2536375_R	antisense	-1,28	11,75
A_19_P00315804	lincRNA:chr9:2530903–2539456_R	antisense	-1,15	10,77
A_19_P00320469	lincRNA:chr9:2535671–2536375_R	antisense	-1,27	9,70
A_19_P00811613	lincRNA:chr9:2452800–2552025_R	antisense	-1,17	7,94
A_33_P3397743	LOC100128088	pseudogene	-1,83	7,42
A_19_P00321203	lincRNA:chr6:72126155–72129954_R	lincRNA	-0,92	7,33
A_19_P00322118	lincRNA:chr2:39745746–39826668_F	antisense	-0,86	6,90
A_23_P3552	LOC730092	pseudogene	-0,65	6,50
A_19_P00322220	lincRNA:chr20:37055062–37063887_R	processed_transcript	-0,69	6,28
A_33_P3392460	LOC100128077	processed_transcript	-1,47	6,18
A_19_P00322149	lincRNA:chr6:72126142–72129923_R	lincRNA	-0,91	6,16
A_19_P00317793	lincRNA:chr20:37055062–37063916_R	processed_transcript	-0,68	6,10
A_19_P00808846	lincRNA:chr21:17992729–18010729_F	lincRNA	-0,64	6,09
A_19_P00318304	lincRNA:chr20:37050986–37063998_R	processed_transcript	-0,67	5,94
A_19_P00316341	lincRNA:chr7:130600800–130606702_F	lincRNA	-0,86	5,93
A_19_P00316985	lincRNA:chr6:72126162–72129969_R	lincRNA	-0,91	5,93
A_19_P00322967	lincRNA:chr20:37050934–37057222_R	processed_transcript	-0,69	5,54
A_19_P00802098	lincRNA:chr2:3579550–3585150_R	lincRNA	-0,58	5,15
A_24_P756289	SOX2OT	other	-0,86	5,08
A_33_P3613516	LOC254057	antisense	-1,10	4,94
A_19_P00318174	lincRNA:chr2:3579840–3584422_R	lincRNA	-0,73	4,52
A_33_P3287710	chr10:79,686,570–79,689,583	unassigned	-0,67	4,46
A_33_P3405043	LOC100133264	unassigned	-0,72	4,43
A_32_P88349	LOC730256	pseudogene	-0,48	4,36
A_33_P3705884	chr19:28,281,401–28,284,848	lincRNA	-0,89	4,27
A_19_P00321044	lincRNA:chr16:50682543–50683160_F	lincRNA	1,04	6,09
A_19_P00315647	lincRNA:chr11:121899032–121899389_R	other	0,65	5,63
A_32_P63013	LOC283174	unassigned	1,32	5,08
A_32_P47157	LOC92973	unassigned	0,74	4,95
A_19_P00317484	lincRNA:chr3:112315643–112316945_R	lincRNA	0,55	4,11
A_19_P00809440	lincRNA:chr11:133765815–133774297_R	other	1,23	4,06
A_33_P3789382	chr10:65,224,989–65,226,322	antisense	0,57	3,98
A_19_P00321420	lincRNA:chr11:133766329–133767054_R	unassigned	1,34	3,74
A_19_P00332120	lincRNA:chr3:156455706–156471081_R	lincRNA	0,59	3,57
A_19_P00320101	lincRNA:chr11:133767609–133771496_R	other	1,06	3,51
A_19_P00812924	lincRNA:chr11:121895965–121904065_R	other	0,53	3,46
A_19_P00326763	lincRNA:chr3:112308735–112318605_R	lincRNA	0,48	2,73
A_33_P3753757	LOC158402	other	0,53	2,46
A_33_P3393679	LOC645323	lincRNA	0,47	2,24
A_19_P00315649	lincRNA:chr11:121899032–121899389_R	other	0,55	2,23
A_19_P00809838	lincRNA:chrX:100247844–100257469_R	unassigned	0,79	2,20
A_19_P00331576	lincRNA:chr3:114043485–114052926_F	unassigned	0,36	2,00
A_33_P3259557	LOC440104	pseudogene	0,45	1,67
A_19_P00319347	lincRNA:chr2:168149680–168414843_F	lincRNA	0,77	1,67
A_19_P00320212	lincRNA:chr9:114795825–114797203_R	other	0,41	1,63
A_24_P93703	LOC440104	pseudogene	0,40	1,50
A_19_P00318878	lincRNA:chr1:247350513–247352101_R	lincRNA	0,38	1,44
A_19_P00316010	lincRNA:chr17:67547498–67549996_F	lincRNA	0,57	1,43
A_24_P349207	ENST00000380727	pseudogene	0,29	1,31
A_19_P00802064	lincRNA:chr8:2522118–2527693_R	lincRNA	0,38	1,04

**Fig 6:**
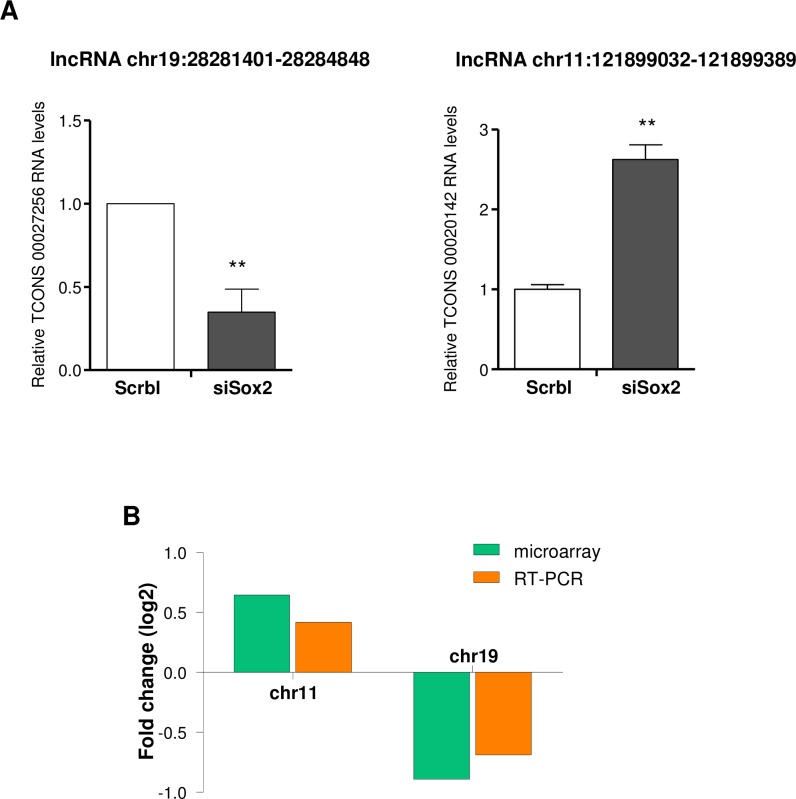
Validation of two lncRNAs regulated by SOX2 in GSC11 cells. (A) The expression of the transcripts located in chr11:121899032–121899389 (TCONS_00020142) and chr19:28,281,401–28,284,848 (TCONS_00027256) were assessed. In both cases GSC11 cells were transfected with siRNA control or siRNA against SOX2 and three days later RNA was extracted and subject to RT-PCR. Values are normalized to GAPDH and are the mean ± SD of three replicates. (B) Comparison between microarray and qRT-PCR results. The height of each column in this graph represents the log-transformed mean fold changes in the expression of lncRNA between Scramble and siSOX2 transfected cell line.

The top four differentially expressed lncRNAs (Table 7) that presented chromatin marks and high abundance in brain were selected using GRCh37/hg19 assembly in UCSC Genome Browser, and their expression was validated using qRT-PCR in GSC11 cells, comparing SOX2-siRNA versus scrambled control. Our data indicated that the expression of chr19:28,281,401–28,284,848 (TCONS_00027256) was significantly down-regulated (p value = 0,018), while chr11:121899032–121899389 (TCONS_00020142) was significantly up-regulated (p value = 0.042) after SOX2 inhibition (Fig 6A). The results were consistent with the microarray data (Fig 6B).

**Table 7:** List of the top-four lncRNAs regulated by Sox2, organized by p-value.

lncRNA	logFC	P.Value	B
lincRNA:chr6:72126155–72129954	-0,9188744	3,40E-07	7,3327381
lincRNA:chr6:29701971–29740296	-0,78724372	3,93E-07	7,2007586
lincRNA: chr19:28,281,401–28,284,848	-0,89247792	8,26E-06	4,265306
lincRNA:chr11:121899032–121899389	0,64633267	2,07E-06	5,6334606

This selection was evaluated by the presence of histone modifications and high abundance in brain according to UCSF genome browser tool.

Altogether, these results identified and confirmed the non-coding transcript profile controlled by SOX2 in GSCs. Characterizing the functional relevance of these lncRNAs will undoubtedly impact our understanding of glioblastoma biology.

## Discussion

Our work provides a comprehensive view of the genome wide SOX2 regulated transcripts in GSCs, illustrating a complex scenario where SOX2 is the central player regulating different molecules and pathways in glioblastoma.

In this study, we used state-of-the art microarray technology to query the SOX2 coding and non-coding RNA transcriptome in GSCs. It is interesting to note that among the down-regulated genes following SOX2 knockdown, F11R has been shown to be overexpressed in glioblastoma cells [42,43]. F11R is necessary and sufficient for GSC maintenance and self-renewal and of clinical significance is associated with increased malignancy and poor patient prognosis [42,43]. On the other hand, we found several interesting over-expressed candidates controlled by SOX2; for example, PPP1R1B is a well-known striatal projection neuron signature marker [44]. The fact that its expression increases following SOX2 inhibition is in line with its role in neuronal differentiation.

In a previous work where the SOX2 response program in a glioblastoma cell line was analyzed [15], authors identified 489 genes whose expression were altered in response to SOX2 knockdown, using several genomic technologies. Interestingly several of these genes are also differentially expressed in our array data, such as NGFR, CEBPA, BCL2, BNIP3, EBF4, ALCAM, protocadherins and solute carrier family members. Overall these results highlight the strength of our array data and the link between SOX2 and GSCs biology. The work of Fang and colleagues exhibits some similarities with our study, such as the analysis of SOX2 regulated-coding genes. However, we focused in the molecular circuitries controlled by SOX2 in GSCs, meanwhile they performed their study in an established glioma cell line. Additionally, our study addressed the SOX2-regulated lncRNAs in GSCs. Altogether both works provide clues regarding SOX2 functions in glioblastoma.

Gene-set enrichment analysis shows SOX2 is involved in regulating “cell adhesion”, “biological adhesion”, “cell-cell signaling”, and “calcium ion binding” pathways, undercovering its key function as a driver of the glioma stem-like phenotype [45–48].

We also analyzed the canonical pathways regulated by SOX2 in GSCs. Pathways related with amino-acid metabolism were among the most deregulated, illustrating that SOX2 expression is critical for maintaining metabolic homeostasis in the GSC population, and plays important role in different tumor microenvironment conditions, such as hypoxic stress conditions [49]. Other enriched pathway altered in our analysis was the NOTCH pathway, where Hes5 and Hey1 had the most significantly down-modulated expression. Hes5 is a marker of neural multipotent progenitors with stem cell properties [50] where it sustains progenitors proliferative state inhibiting their differentiation into neurons [51]. On the other hand Hey1 has been related to a subset of molecules directly associated with hypoxia in glioblastoma tumors [52]; and might be used as a marker to distinguish glioblastoma patients with a relative good prognosis (negative Hey1 expression) [53]. Furthermore, Hey 1 is up-regulated in glioma samples correlating with tumor grade, and functionally its down-regulation results in a proliferation reduction [54], suggesting a role in the progression of glioblastoma. Taking all this into account, the canonical pathways more significantly altered after SOX2 inhibition are those related with intracellular signaling cascades and amino-acid metabolism pathways associated with tumor propagation.

Consistent with what is known about SOX2 biological function, our data-set is enriched with genes involved in morphology determination, development and cellular proliferation and migration. Interestingly, “proliferation of tumor cells” (S2 Table), is one of the most repeated subcategories for which IPA analysis assigned an activation Z score close to -2, predicting it´s inhibition, which is in line with a putative role of SOX2 in cell proliferation.

One of the most exciting aspects of our study involved expanding our knowledge of the SOX2 transcriptome into the realm of lncRNAs. In this study we showed and classified the lncRNA landscape regulated by SOX2 in GSCs. Our microarray results showed a strong correlation with published reports, demonstrating the strength of our approach and providing confidence that we can use this data-set for de novo discovery of novel SOX2 targets, including lncRNAs. One previous study determined the differentially expressed lncRNAs between glioblastoma and brain tissues, showing 654 lncRNAs upregulated and 654 down-regulated [55]. To our knowledge, this is the first study that evaluates the differentially expression of lncRNAs in GSCs controlled by SOX2.

Among the transcripts regulated by SOX2 we found that SOX2OT was down-regulated in our data set, even though we did not validate it. SOX2OT is a lncRNA which harbors SOX2 gene in its intronic region and is transcribed in the same orientation as SOX2 [56]. Several studies have demonstrated a role of SOX2OT in the regulation of SOX2 gene in human stem cells [57,58] although little is known about the exact role of this non-coding RNA. SOX2OT has been associated with carcinogenesis and, for example in breast cancer is involved in the induction and/or maintenance of SOX2 expression [59], in esophageal squamous cell carcinoma has been shown to play a role in tumor initiation and/or progression as well as in regulation of the pluripotent state of stem cells [58], and proliferation in lung cancer cells [60]. Askarian-Amiri et al demonstrated that SOX2OT has a positive effect on SOX2 expression [59]. Published data suggest the mediation of lncRNA SOX2OT in pluripotency and tumorigenesis events, probably through regulation of SOX2 expression. These data together with our own results suggest a possible role of SOX2OT in the malignant phenotype of glioblastoma, however further functional and mechanistic studies will be necessary to elucidate the precise role of SOX2OT and other lncRNA candidates in the tumorigenicity of glioblastoma.

Although advance in managing and treating glioblastoma have been made, tumor recurrence and treatment resistance remains the major cause of glioblastoma mortality. Understanding how factors such as SOX2 drive the glioblastoma tumor phenotype will aid in the development of new therapeutic approaches based on targeting GSCs. Our study integrates for the first time the coding and non-coding transcriptome controlled by SOX2 in GSCs, gaining new insights about the molecular circuitries governing glioblastoma biology.

## Conclusion

In conclusion we have performed a comprehensive analysis of differential expression of coding and non-coding transcripts controlled by SOX2 in GSCs. We performed gene set enrichment analysis to find the most relevant pathways and biological functions altered in our data set. This integrated analysis allows for a better understanding of the SOX2 transcriptome in GSCs.

## Supporting Information

S1 Table Differentially expressed coding transcripts from microarray data. (XLS)

S2 Table List of disease and biofunctions data from Ingenuity analysis. (XLS)

## References

[1] Ostrom QT, Gittleman H, Stetson L, Virk SM, Barnholtz-Sloan JS. Epidemiology of gliomas. Cancer Treat Res. 2015;163: 1–14. 10.1007/978-3-319-12048-5_1 25468222

[2] Dolecek TA, Propp JM, Stroup NE, Kruchko C. CBTRUS statistical report: primary brain and central nervous system tumors diagnosed in the United States in 2005–2009. Neuro Oncol. 2012;14 Suppl 5: v1–49. 10.1093/neuonc/nos218 23095881 PMC3480240

[3] Stupp R, Hegi ME, Mason WP, van den Bent MJ, Taphoorn MJ, Janzer RC, et al Effects of radiotherapy with concomitant and adjuvant temozolomide versus radiotherapy alone on survival in glioblastoma in a randomised phase III study: 5-year analysis of the EORTC-NCIC trial. Lancet Oncol. 2009;10: 459–466. 10.1016/S1470-2045(09)70025-7 19269895

[4] Singh SK, Hawkins C, Clarke ID, Squire JA, Bayani J, Hide T, et al Identification of human brain tumour initiating cells. Nature. 2004;432: 396–401. 15549107 10.1038/nature03128

[5] Singh AK, Arya RK, Maheshwari S, Singh A, Meena S, Pandey P, et al Tumor heterogeneity and cancer stem cell paradigm: updates in concept, controversies and clinical relevance. Int J Cancer. 2015;136: 1991–2000. 10.1002/ijc.28804 24615680

[6] Wakimoto H, Mohapatra G, Kanai R, Curry WT Jr, Yip S, Nitta M, et al Maintenance of primary tumor phenotype and genotype in glioblastoma stem cells. Neuro Oncol. 2012;14: 132–144. 10.1093/neuonc/nor195 22067563 PMC3266381

[7] Bao S, Wu Q, McLendon RE, Hao Y, Shi Q, Hjelmeland AB, et al Glioma stem cells promote radioresistance by preferential activation of the DNA damage response. Nature. 2006;444: 756–760. 17051156 10.1038/nature05236

[8] Auffinger B, Spencer D, Pytel P, Ahmed AU, Lesniak MS. The role of glioma stem cells in chemotherapy resistance and glioblastoma multiforme recurrence. Expert Rev Neurother. 2015;15: 741–752. 10.1586/14737175.2015.1051968 26027432 PMC4830899

[9] Avilion AA, Nicolis SK, Pevny LH, Perez L, Vivian N, Lovell-Badge R. Multipotent cell lineages in early mouse development depend on SOX2 function. Genes Dev. 2003;17: 126–140. 12514105 10.1101/gad.224503 PMC195970

[10] Hussenet T, Dali S, Exinger J, Monga B, Jost B, Dembele D, et al SOX2 is an oncogene activated by recurrent 3q26.3 amplifications in human lung squamous cell carcinomas. PLOS ONE. 2010;5: e8960 10.1371/journal.pone.0008960 20126410 PMC2813300

[11] Bass AJ, Watanabe H, Mermel CH, Yu S, Perner S, Verhaak RG, et al SOX2 is an amplified lineage-survival oncogene in lung and esophageal squamous cell carcinomas. Nat Genet. 2009;41: 1238–1242. 10.1038/ng.465 19801978 PMC2783775

[12] Ben-Porath I, Thomson MW, Carey VJ, Ge R, Bell GW, Regev A, et al An embryonic stem cell-like gene expression signature in poorly differentiated aggressive human tumors. Nat Genet. 2008;40: 499–507. 10.1038/ng.127 18443585 PMC2912221

[13] Alonso MM, Diez-Valle R, Manterola L, Rubio A, Liu D, Cortes-Santiago N, et al Genetic and epigenetic modifications of Sox2 contribute to the invasive phenotype of malignant gliomas. PLOS ONE. 2011;6: e26740 10.1371/journal.pone.0026740 22069467 PMC3206066

[14] Gangemi RM, Griffero F, Marubbi D, Perera M, Capra MC, Malatesta P, et al SOX2 silencing in glioblastoma tumor-initiating cells causes stop of proliferation and loss of tumorigenicity. Stem Cells. 2009;27: 40–48. 10.1634/stemcells.2008-0493 18948646

[15] Fang X, Yoon JG, Li L, Yu W, Shao J, Hua D, et al The SOX2 response program in glioblastoma multiforme: an integrated ChIP-seq, expression microarray, and microRNA analysis. BMC Genomics. 2011;12: 11-2164-12-11. 10.1186/1471-2164-12-11 PMC3022822 21211035

[16] Mercer TR, Dinger ME, Mattick JS. Long non-coding RNAs: insights into functions. Nat Rev Genet. 2009;10: 155–159. 10.1038/nrg2521 19188922

[17] Ying L, Huang Y, Chen H, Wang Y, Xia L, Chen Y, et al Downregulated MEG3 activates autophagy and increases cell proliferation in bladder cancer. Mol Biosyst. 2013;9: 407–411. 10.1039/c2mb25386k 23295831

[18] Gupta RA, Shah N, Wang KC, Kim J, Horlings HM, Wong DJ, et al Long non-coding RNA HOTAIR reprograms chromatin state to promote cancer metastasis. Nature. 2010;464: 1071–1076. 10.1038/nature08975 20393566 PMC3049919

[19] Zhou Y, Zhang X, Klibanski A. MEG3 noncoding RNA: a tumor suppressor. J Mol Endocrinol. 2012;48: R45–53. 10.1530/JME-12-0008 22393162 PMC3738193

[20] Hauptman N, Glavac D. Long non-coding RNA in cancer. Int J Mol Sci. 2013;14: 4655–4669. 10.3390/ijms14034655 23443164 PMC3634483

[21] Gibb EA, Vucic EA, Enfield KS, Stewart GL, Lonergan KM, Kennett JY, et al Human cancer long non-coding RNA transcriptomes. PLOS ONE. 2011;6: e25915 10.1371/journal.pone.0025915 21991387 PMC3185064

[22] Zhang XQ, Sun S, Lam KF, Kiang KM, Pu JK, Ho AS, et al A long non-coding RNA signature in glioblastoma multiforme predicts survival. Neurobiol Dis. 2013;58: 123–131. 10.1016/j.nbd.2013.05.011 23726844

[23] Smyth GK. Linear models and empirical bayes methods for assessing differential expression in microarray experiments. Stat Appl Genet Mol Biol. 2004;3: Article3. 10.2202/1544-6115.1027 16646809

[24] Gentleman R, Carey V, Dudoit S, Irizarry R, Huber W. Bioinformatics and computational biology solutions using R and Bioconductor Gentleman R, Carey V, Huber W, Irizarry R, Dudoit S. ed. New York, NY: Springer; 2005.

[25] Nuutinen S, Panula P. Histamine in neurotransmission and brain diseases. Adv Exp Med Biol. 2010;709: 95–107. 21618891 10.1007/978-1-4419-8056-4_10

[26] Chen L, Li X, Liu L, Yu B, Xue Y, Liu Y. Erastin sensitizes glioblastoma cells to temozolomide by restraining xCT and cystathionine-gamma-lyase function. Oncol Rep. 2015;33: 1465–1474. 10.3892/or.2015.3712 25585997

[27] Dinan TG. Serotonin: current understanding and the way forward. Int Clin Psychopharmacol. 1996;11 Suppl 1: 19–21. 8732440

[28] Yang S, Li WS, Dong F, Sun HM, Wu B, Tan J, et al KITLG is a novel target of miR-34c that is associated with the inhibition of growth and invasion in colorectal cancer cells. J Cell Mol Med. 2014;18: 2092–2102. 10.1111/jcmm.12368 25213795 PMC4244023

[29] Carson WE, Haldar S, Baiocchi RA, Croce CM, Caligiuri MA. The c-kit ligand suppresses apoptosis of human natural killer cells through the upregulation of bcl-2. Proc Natl Acad Sci U S A. 1994;91: 7553–7557. 7519782 10.1073/pnas.91.16.7553 PMC44440

[30] Flanagan JG, Chan DC, Leder P. Transmembrane form of the kit ligand growth factor is determined by alternative splicing and is missing in the Sld mutant. Cell. 1991;64: 1025–1035. 1705866 10.1016/0092-8674(91)90326-t

[31] Lea RW, Dawson T, Martinez-Moreno CG, El-Abry N, Harvey S. Growth hormone and cancer: GH production and action in glioma? Gen Comp Endocrinol. 2015;220: 119–123. 10.1016/j.ygcen.2015.06.011 26163024

[32] Minchenko DO, Kharkova AP, Hubenia OV, Minchenko OH. Insulin receptor, IRS1, IRS2, INSIG1, INSIG2, RRAD, and BAIAP2 gene expressions in glioma U87 cells with ERN1 loss of function: effect of hypoxia and glutamine or glucose deprivation. Endocr Regul. 2013;47: 15–26. 23363253 10.4149/endo_2013_01_15

[33] Wang J, Wakeman TP, Lathia JD, Hjelmeland AB, Wang XF, White RR, et al Notch promotes radioresistance of glioma stem cells. Stem Cells. 2010;28: 17–28. 10.1002/stem.261 19921751 PMC2825687

[34] Fan X, Khaki L, Zhu TS, Soules ME, Talsma CE, Gul N, et al NOTCH pathway blockade depletes CD133-positive glioblastoma cells and inhibits growth of tumor neurospheres and xenografts. Stem Cells. 2010;28: 5–16. 10.1002/stem.254 19904829 PMC2878196

[35] Gilbert CA, Daou MC, Moser RP, Ross AH. Gamma-secretase inhibitors enhance temozolomide treatment of human gliomas by inhibiting neurosphere repopulation and xenograft recurrence. Cancer Res. 2010;70: 6870–6879. 10.1158/0008-5472.CAN-10-1378 20736377 PMC2932884

[36] Zhou HY, Katsman Y, Dhaliwal NK, Davidson S, Macpherson NN, Sakthidevi M, et al A Sox2 distal enhancer cluster regulates embryonic stem cell differentiation potential. Genes Dev. 2014;28: 2699–2711. 10.1101/gad.248526.114 25512558 PMC4265674

[37] Ferri A, Favaro R, Beccari L, Bertolini J, Mercurio S, Nieto-Lopez F, et al Sox2 is required for embryonic development of the ventral telencephalon through the activation of the ventral determinants Nkx2.1 and Shh. Development. 2013;140: 1250–1261. 10.1242/dev.073411 23444355

[38] Dinger ME, Amaral PP, Mercer TR, Pang KC, Bruce SJ, Gardiner BB, et al Long noncoding RNAs in mouse embryonic stem cell pluripotency and differentiation. Genome Res. 2008;18: 1433–1445. 10.1101/gr.078378.108 18562676 PMC2527704

[39] Dey BK, Mueller AC, Dutta A. Long non-coding RNAs as emerging regulators of differentiation, development, and disease. Transcription. 2014;5: e944014 10.4161/21541272.2014.944014 25483404 PMC4581346

[40] Zhu LJ, Gazin C, Lawson ND, Pages H, Lin SM, Lapointe DS, et al ChIPpeakAnno: a Bioconductor package to annotate ChIP-seq and ChIP-chip data. BMC Bioinformatics. 2010;11: 237-2105-11-237. 10.1186/1471-2105-11-237 PMC3098059 20459804

[41] Harrow J, Frankish A, Gonzalez JM, Tapanari E, Diekhans M, Kokocinski F, et al GENCODE: the reference human genome annotation for The ENCODE Project. Genome Res. 2012;22: 1760–1774. 10.1101/gr.135350.111 22955987 PMC3431492

[42] Lathia JD, Li M, Sinyuk M, Alvarado AG, Flavahan WA, Stoltz K, et al High-throughput flow cytometry screening reveals a role for junctional adhesion molecule a as a cancer stem cell maintenance factor. Cell Rep. 2014;6: 117–129. 10.1016/j.celrep.2013.11.043 24373972 PMC3899718

[43] Alvarado AG, Turaga SM, Sathyan P, Mulkearns-Hubert EE, Otvos B, Silver DJ, et al Coordination of self-renewal in glioblastoma by integration of adhesion and microRNA signaling. Neuro Oncol. 2016;18: 656–666. 10.1093/neuonc/nov196 26374689 PMC4827035

[44] Arber C, Precious SV, Cambray S, Risner-Janiczek JR, Kelly C, Noakes Z, et al Activin A directs striatal projection neuron differentiation of human pluripotent stem cells. Development. 2015;142: 1375–1386. 10.1242/dev.117093 25804741 PMC4378247

[45] Inoue A, Takahashi H, Harada H, Kohno S, Ohue S, Kobayashi K, et al Cancer stem-like cells of glioblastoma characteristically express MMP-13 and display highly invasive activity. Int J Oncol. 2010;37: 1121–1131. 20878060 10.3892/ijo_00000764

[46] Reddy EM, Chettiar ST, Kaur N, Ganeshkumar R, Shepal V, Shanbhag NC, et al Dlxin-1, a member of MAGE family, inhibits cell proliferation, invasion and tumorigenicity of glioma stem cells. Cancer Gene Ther. 2011;18: 206–218. 10.1038/cgt.2010.71 21109781

[47] Dietrich J, Diamond EL, Kesari S. Glioma stem cell signaling: therapeutic opportunities and challenges. Expert Rev Anticancer Ther. 2010;10: 709–722. 10.1586/era.09.190 20470003

[48] Liebelt BD, Shingu T, Zhou X, Ren J, Shin SA, Hu J. Glioma Stem Cells: Signaling, Microenvironment, and Therapy. Stem Cells Int. 2016;2016: 7849890 10.1155/2016/7849890 26880988 PMC4736567

[49] Kucharzewska P, Christianson HC, Belting M. Global profiling of metabolic adaptation to hypoxic stress in human glioblastoma cells. PLOS ONE. 2015;10: e0116740 10.1371/journal.pone.0116740 25633823 PMC4310608

[50] Basak O, Taylor V. Identification of self-replicating multipotent progenitors in the embryonic nervous system by high Notch activity and Hes5 expression. Eur J Neurosci. 2007;25: 1006–1022. 17331197 10.1111/j.1460-9568.2007.05370.x

[51] Ross SE, Greenberg ME, Stiles CD. Basic helix-loop-helix factors in cortical development. Neuron. 2003;39: 13–25. 12848929 10.1016/s0896-6273(03)00365-9

[52] Irshad K, Mohapatra SK, Srivastava C, Garg H, Mishra S, Dikshit B, et al A combined gene signature of hypoxia and notch pathway in human glioblastoma and its prognostic relevance. PLOS ONE. 2015;10: e0118201 10.1371/journal.pone.0118201 25734817 PMC4348203

[53] Gaetani P, Hulleman E, Levi D, Quarto M, Scorsetti M, Helins K, et al Expression of the transcription factor HEY1 in glioblastoma: a preliminary clinical study. Tumori. 2010;96: 97–102. 20437865 10.1177/030089161009600116

[54] Hulleman E, Quarto M, Vernell R, Masserdotti G, Colli E, Kros JM, et al A role for the transcription factor HEY1 in glioblastoma. J Cell Mol Med. 2009;13: 136–146. 10.1111/j.1582-4934.2008.00307.x 18363832 PMC3823042

[55] Han L, Zhang K, Shi Z, Zhang J, Zhu J, Zhu S, et al LncRNA pro fi le of glioblastoma reveals the potential role of lncRNAs in contributing to glioblastoma pathogenesis. Int J Oncol. 2012;40: 2004–2012. 10.3892/ijo.2012.1413 22446686

[56] Fantes J, Ragge NK, Lynch SA, McGill NI, Collin JR, Howard-Peebles PN, et al Mutations in SOX2 cause anophthalmia. Nat Genet. 2003;33: 461–463. 12612584 10.1038/ng1120

[57] Amaral PP, Neyt C, Wilkins SJ, Askarian-Amiri ME, Sunkin SM, Perkins AC, et al Complex architecture and regulated expression of the Sox2ot locus during vertebrate development. RNA. 2009;15: 2013–2027. 10.1261/rna.1705309 19767420 PMC2764477

[58] Shahryari A, Rafiee MR, Fouani Y, Oliae NA, Samaei NM, Shafiee M, et al Two novel splice variants of SOX2OT, SOX2OT-S1, and SOX2OT-S2 are coupregulated with SOX2 and OCT4 in esophageal squamous cell carcinoma. Stem Cells. 2014;32: 126–134. 10.1002/stem.1542 24105929

[59] Askarian-Amiri ME, Seyfoddin V, Smart CE, Wang J, Kim JE, Hansji H, et al Emerging role of long non-coding RNA SOX2OT in SOX2 regulation in breast cancer. PLOS ONE. 2014;9: e102140 10.1371/journal.pone.0102140 25006803 PMC4090206

[60] Hou Z, Zhao W, Zhou J, Shen L, Zhan P, Xu C, et al A long noncoding RNA Sox2ot regulates lung cancer cell proliferation and is a prognostic indicator of poor survival. Int J Biochem Cell Biol. 2014;53: 380–388. 10.1016/j.biocel.2014.06.004 24927902

